# P-291. College PrEP: Impacts of Targeted Education on Awareness and Engagement in HIV Prevention Services Among Adolescents and Young Adults (AYA)

**DOI:** 10.1093/ofid/ofaf695.512

**Published:** 2026-01-11

**Authors:** Errol Fields, Kelly E Pillinger, Leah Molloy, Laura Simone, Chris Napolitan, Jenniffer A Meza Jimenez, Jeffrey D Carter, Bonnie Douglas

**Affiliations:** Johns Hopkins University, Baltimore, MD; PRIME Education, New York, NY; PRIME Education, New York, NY; PRIME Education, LLC, Fort Lauderdale, Florida; PRIME Education, New York, NY; PRIME Education, New York, NY; PRIME Education, LLC, Fort Lauderdale, Florida; PRIME Education, LLC, Fort Lauderdale, Florida

## Abstract

**Background:**

AYA make up 1 in 5 new HIV diagnoses and have lower testing rates than adults. This project aimed to identify discordances between AYA-reported and provider-perceived barriers to HIV prevention and support engagement in prevention services among AYA in high school/college.

**Table 1.**
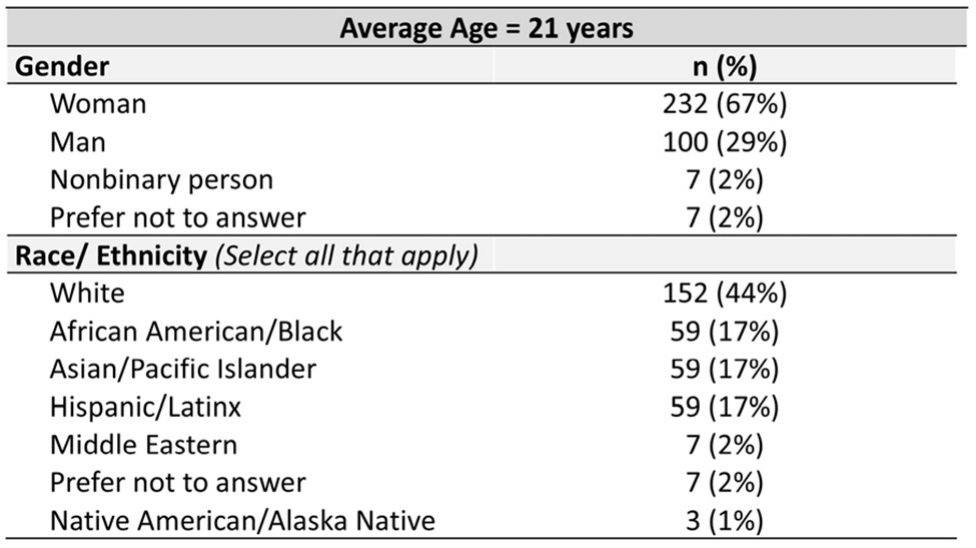
Demographics of Student Participants in Education Sessions (N = 346).

**Table 2.**
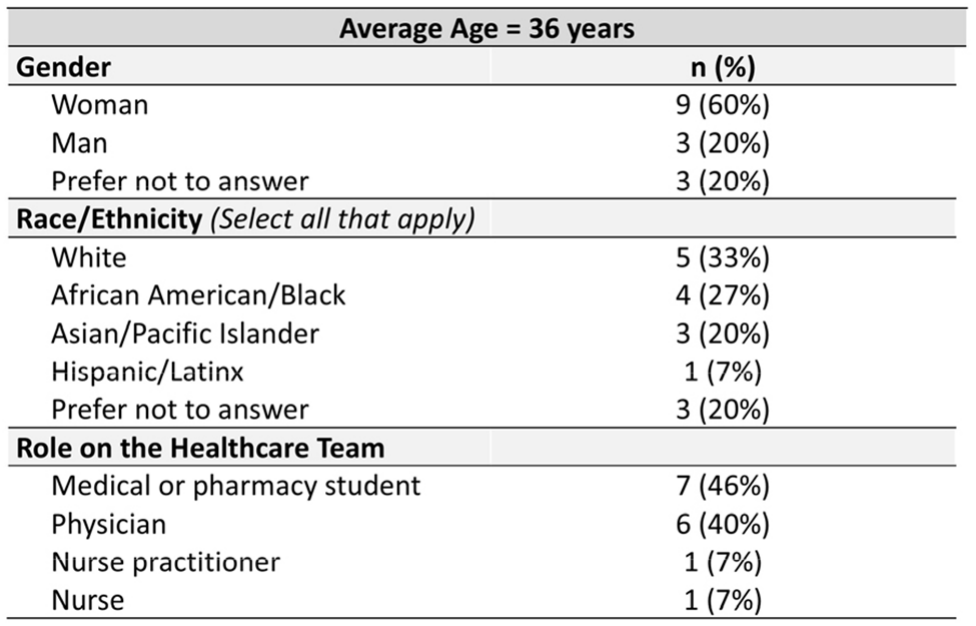
Demographics of HCP Participants in Education Sessions (N = 15).

**Methods:**

One-hour education sessions were led virtually or in-person by healthcare professionals and trainees (HCPs) with students at 7 universities and 1 high school in the Southeast US and California (2/2024 – 3/2025). Students and HCPs completed pre- and post-surveys at sessions. Follow-up surveys were sent out 90 days after sessions to assess long-term impacts. Data were analyzed using a Chi-square test.

**Figure 1.**
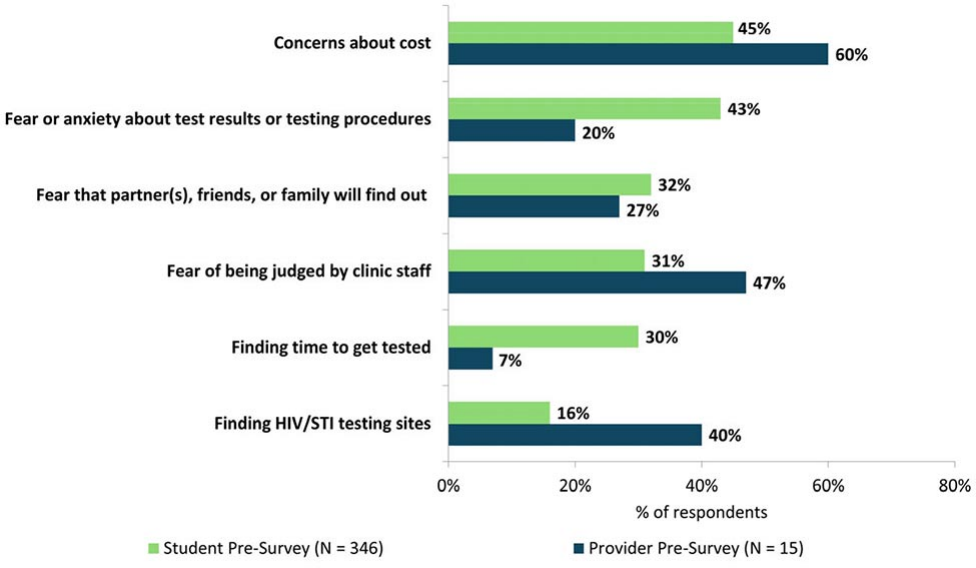
Student self-reported and HCP-perceived challenges to getting tested for HIV and sexually transmitted infections (STIs).

**Figure 2.**
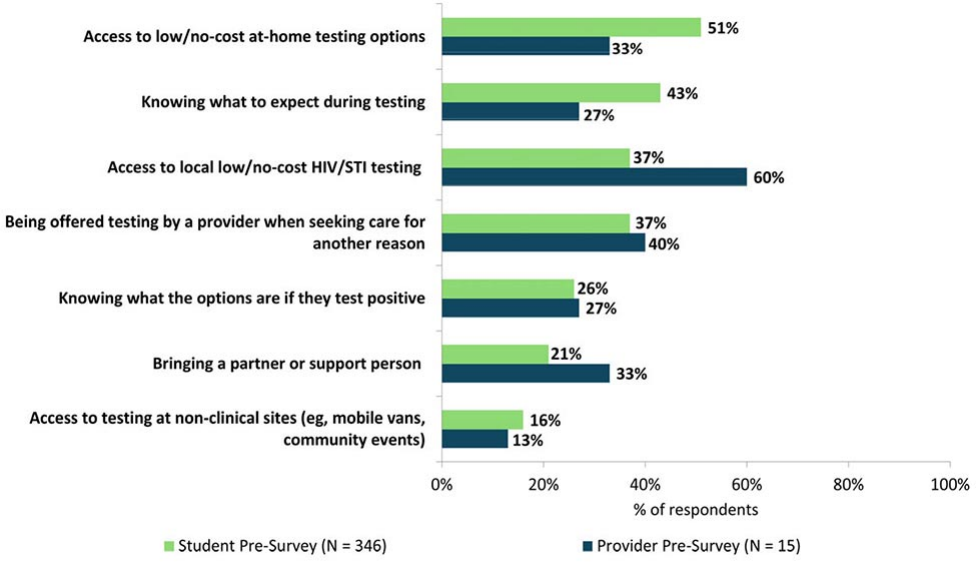
Student self-reported and HCP-perceived facilitators to HIV/STI testing.

**Results:**

In total, 346 students and 15 HCPs attended sessions (Tables 1, 2). On pre-session surveys, only 23% of students had been HIV tested and 8% had discussed pre-exposure prophylaxis (PrEP) with a provider. When asked what they perceived as students’ top barriers to HIV/STI testing, HCPs noted cost, fear of judgment in clinics, and finding testing sites. Students reported cost but also anxiety about test procedures/results and fear that others would find out they were tested (Fig 1). When asked what would make testing easier, both groups noted local low/no-cost testing, but students prioritized at-home testing and knowing what to expect during tests (Fig 2). Students said side effects (49%) and cost/insurance coverage (44%) were top barriers to taking PrEP. Student knowledge and confidence improved on post-session surveys vs pre-session: higher proportions reported high knowledge of PrEP options (64% vs 9%, p < .0001), agreed they were aware of campus resources for sexual health (95% vs 54%, p < .0001), and were likely to get tested for HIV (42% vs 18%, p < .0001). Of students responding to follow-up surveys (N = 113), 41% had been tested for HIV and 66% had spoken to an HCP about HIV prevention or made an appointment to since the session. HCPs responding to follow-up surveys (N = 10) had helped > 100 AYA get tested for HIV since sessions, and 90% were likely to lead another session.

**Conclusion:**

These findings reveal differences in HCP-perceived and AYA-reported barriers/facilitators to HIV prevention, highlighting areas for improvement in patient counseling and the utility of HCP-led campus-based education to reach AYA.

**Disclosures:**

All Authors: No reported disclosures

